# Contrasting microbiota profiles observed in children carrying either *Blastocystis* spp. or the commensal amoebas *Entamoeba coli* or *Endolimax nana*

**DOI:** 10.1038/s41598-020-72286-y

**Published:** 2020-09-18

**Authors:** Juan F. Alzate, Miguel Toro-Londoño, Felipe Cabarcas, Gisela Garcia-Montoya, Ana Galvan-Diaz

**Affiliations:** 1 Departamento de Microbiología Y Parasitología, Facultad de Medicina, Universidad de Antioquia, Medellín, Colombia; 2 Facultad de Medicina, Centro Nacional de Secuenciación genómica - CNSG, Sede de Investigación Universitaria - SIU, Universidad de Antioquia, Medellín, Colombia; 3 Facultad de Ingeniería, Grupo Sistemic, Universidad de Antioquia, Medellín, Colombia; 4 Grupo de Microbiología Ambiental, Escuela de Microbiología, Universidad de Antioquia, Medellín, Colombia

**Keywords:** Microbiome, Parasite host response

## Abstract

Recent studies have shown how intestinal parasites can modulate gut microbiota. This observation is not surprising since the human intestinal lumen, like any other niche, is a battlefield of microbial competition, and Eukaryotes can affect bacterial populations. Intestinal pathogenic protist has been associated with reshaping the microbial community structure; however, the interactions between the colonic bacterial communities and parasites like *Blastocystis* spp., *Entamoeba coli*, and *Endolimax nana* have been poorly studied. In this work, we studied the distal intestinal bacterial microbiota of 49 children attending 7 public daycare centers in Medellin, Colombia, and compared the bacterial microbiota structure in the presence or absence of the protists *Blastocystis* spp., *E. coli*, and *E. nana*. Parasite colonization was associated with an increase in bacterial richness. Moreover, *Blastocystis* spp. presented a positive relationship with *Prevotella*, since this bacterium was selectively enriched in children carrying it. Remarkably, the *E. coli* colonized children showed a microbial profile that was closer to uninfected controls, although some bacterial taxa displayed to be enriched. This is the case for *Akkermansia*, which showed to be favored in *E. coli* colonized individuals, while notably reduced in the *Blastocystis* spp. parasitized group.

## Introduction

A complex microbial community, mostly bacteria, colonizes human intestines since birth, although it also bears archaea, fungi, viruses and occasionally parasites. Recent research works have confirmed that under normal conditions, human adults can harbor between 500 to 1,000 different species of bacteria that along with its metabolic products can influence the health state of the organism^1^. These abundant microbes can help us processing food, absorb vitamins, stimulate the immune system^2,3^ and compete with intestinal pathogens, amid other functions^4–6^.

In early childhood, the offspring microbiota is strongly associated with the mother’s and with the delivery mode. This will influence if the colonizing bacteria mainly come from skin, vagina, or even stool. As the child grows and solid foods are introduced, new microbes arrive increasing the diversity and favoring bacteria capable of degrading complex polymers. It is estimated that diversity becomes stable, adult-like, at around 2.5 years old^7,8^. By this age two Phyla dominate the microbiota of healthy individuals: Bacteroidetes and Firmicutes^9,10^.

It has been extensively described that the main driving force for the intestinal microbial structure is the diet^2,6,11–14^. Other factors such as host genetics^15^, sex^16^, and antibiotic use might also have a significant influence in the community shaping as well^17–19^.

Other prominent players in the gut microbiome competition are parasites, which gained attention in recent years. Several works have shown how microbiota richness and community structure is influenced by protist and metazoan parasites. Human intestinal nematodes have been described to be associated with increased microbial richness and diversity^20–23^, whereas intestinal protist have shown a broader panorama, where interactions with the intestinal microbiota depend on the species studied^24^. One of the most studied models is *Entamoeba histolytica,* in which specific bacterial taxa are disease predictors. Patients with diarrhea and positive for *E. histolytica* had higher levels of *Prevotella copri*, compared to nondiarrheal subjects^25^. Moreover, it was demonstrated that trophozoites of this parasite phagocyte bacteria like *Lactobacillus* and *Shigella dysenteriae* conferring an augmented cytopathic effect to the parasite^26,27^.

Another studied case is *Blastocystis* spp. which has been associated in several studies with a bacterial species richness increase in the distal intestine^28–30^. *Blastocystis* is an anaerobic Stramenopila that inhabits the gastrointestinal tract of a wide range of animal hosts, including humans. It is one of the most frequent intestinal protists worldwide colonizing around 1 billion people, being associated with an oral-fecal route of transmission. There are reports of a high prevalence of this protist in both developing (up to 100%)^31^ and developed countries (0.5–30%)^32–34^. In Colombia, studies from 2009–2019 describe a prevalence between 12.6 and 87.1% in children^35,36^, which are the age group at a higher risk of intestinal parasite infection^34^.

Based on the sequence analysis of the SSU rDNA gene, to date 17 *Blastocystis* subtypes (STs) have already been identified (ST1-ST17), and genetic diversity studies have shown intra-genetic diversity among STs^37–39^. Subtypes ST1-ST9 and ST12 parasite humans; being STs 1–4 the most prevalent. ST9 has been reported exclusively on human hosts. Differences in the development of symptoms and their severity have been described (abdominal pain, constipation, diarrhea, flatulence, irritable bowel syndrome-like symptoms-IBS, and even skin disorders such as urticaria)^34,40,41^. One reason that could explain this broad spectrum of reactions can be attributed to the genetic diversity described in this parasite.

Several studies have reported a *Blastocystis* spp. long-term colonization in asymptomatic carriers, suggesting that it could be a frequent member of the healthy intestinal microbiota^42–44^. Recent data about the relationship between this protist and the gut bacteria supports the latter hypothesis. Most of the research, performed mainly in the developed world, shows a higher fecal bacterial richness and diversity in individuals carrying the parasite^29,30^. In the developing world, the same phenomenon was observed, where adults in Mexico (Morelos) positive to *Blastocystis* spp. showed increased bacterial diversity compared with not infected ones^28^. Besides, this protist is associated with gut microbiota profiles characterized by low relative abundances of *Bacteroides*-driven enterotype and high levels of *Ruminococcus*- and *Prevotella*-driven enterotype, taxa that are typically associated with gut health^28,45–49^. A eubiotic condition was also associated with *Blastocystis* spp. since a significantly higher ratio of beneficial species (*Faecalibacterium prausnitzii)* versus potentially harmful species (*Escherichia coli*) was found in individuals positive for this parasite^47^.

Additionally, some authors described a lower abundance of potentially pathogenic species of the *Enterobacteriaceae* family in the presence of *Blastocystis* spp. and *Entamoeba* compared to negative controls^47^. On the contrary, a few studies describe a dysbiosis in patients with *Blastocystis* spp. Nourrisson et al.^50^ found lower protective bacteria in the fecal microbiota in patients with irritable bowel syndrome and healthy controls. Yason et al. described that mice infected with *Blastocystis* ST7 had less beneficial *Bifidobacterium* and *Lactobacillus* bacteria. Microbial communities are probably differently shaped according to the *Blastocystis* subtypes^51^.

Most of the *Blastocystis* spp. and intestinal microbiota interaction studies have been done in the adult population. Data regarding gut bacteria and *Blastocystis* spp. interactions in children are scarce. Popovic et al.^49^ characterized the eukaryotic microbiota of hospitalized Malawian children suffering from Severe Acute Malnutrition (SAM). *Blastocystis* colonization correlated with bacterial alpha diversity and increased abundance of specific taxa from Firmicutes, particularly those from the clostridia class (*Oscillibacter*, *Sporobacter*, *Cellulosibacter*, and *Roseburia*). Consistent with the results of previous studies, there was a negative correlation between *Blastocystis* and the *Enterobacteriaceae* family. In Colombia, Toro et al.^52^ evaluated the bacterial gut microbiota composition in children infected with intestinal parasites. Four groups were included according to the parasites identified. Children from the *Giardia* group (only infected with *Giardia*) and Helm-pro group (infected with nematode and another protist including *Blastocystis*) suffer a switch from a type I (*Bacteroides*-driven enterotype) to a type II enterotype (*Prevotella*-driven enterotype).

In this work, we study the alterations of the gut microbiota on children from one to five years old colonized by the protists *Blatocystis* spp., *Entamoeba coli*, and *Endolimax nana*. Albeit the intense research of the gut microbiota, still, little information is available on the effect of these parasites in children and, in the case of *E. coli* and *E. nana*, information considering this topic is scarce.

## Results

The 16S metataxonomic experiment started with 50 k reads per library. After MOTHUR processing, the read number was rarefied with the *totalgroup* function to an average of 12,249 clean reads per sample, and they ranged between 12,326 and 12,526. The coverage analysis showed that at least 97.4% of the expected OTUs were observed within our analysis individuals and went up to 99% in several samples. The observed OTU (3%) count varied between 193 and 551 within all tested individuals (Supplementary Table 1).

### Effect of harboring protists

**Figure 1:**
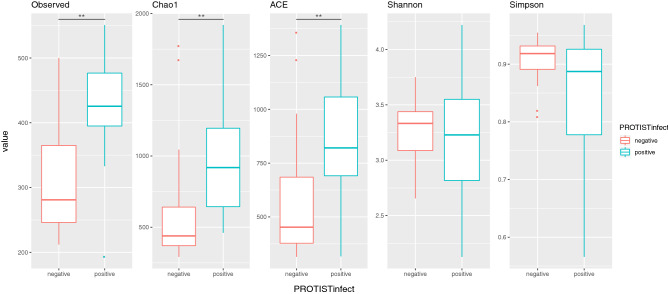
Richness and diversity indices of protist infected and control children. Boxplot representation of the median values of the Observed OTUs and the Chao1, ACE, Shannon, and Simpson indices; comparing the control children (no parasites detectable on the stool analysis—**negative**) and the Protist infected group (**positive**: colonized either with *Blastocystis spp., Entamoeba coli*, or *Endolimax nana*). The observed differences in the median values between the Protist negative and positive groups for the observed OTUs, Chao1 index, and ACE index, were statistically significant with p < 0.001 (Kruskal–Wallis rank-sum test).

Initially, we tested the differences between children with no detectable parasites on the stool microscopical analysis (NPDM group) and those confirmed to harbor either *Blastocystis spp.* (Blasto group), *Entamoeba coli* (E_coli group) or *Endolimax nana* (E_nana); these last three were grouped in the PROTIST-infected category. As shown in Fig. 1, the Observed OTU median values rise from 281 in NPDM controls (negative) to 426 in the protist-infected ones (positive), with a p-value = 3.785e-05, using the Kruskal–Wallis rank-sum test. Similarly, the Chao1 and ACE indices were significantly higher, using the same test, in infected individuals compared to controls, rising from 439 to 919 (p-value = 0.0001335), and from 453 to 821 (p-value = 0.0003182), respectively. The Shannon and Simpson indices for both groups were similar and showed no statistically significant differences with the Kruskal–Wallis test. Furthermore, sex did not show any significant difference with the richness/diversity indices studied, when the Kruskal–Wallis test was applied.

Regarding age, there was a weak significative correlation with the Shannon index (Spearman correlation p-value = 0.02515, rho 0.3196735), which was not the case for the Simpson index, that showed no significant results. The ‘lm’ model of Age and Shannon gives shannon = 0.1Age + 2.8 with (p-value =  < 2e−16, for the intercept and p-value = 0.0596 for the slope). Our group of selected children, most are in the range of 2 to 5yo, life period in which the intestinal microbiota tends to stabilize.

**Figure 2:**
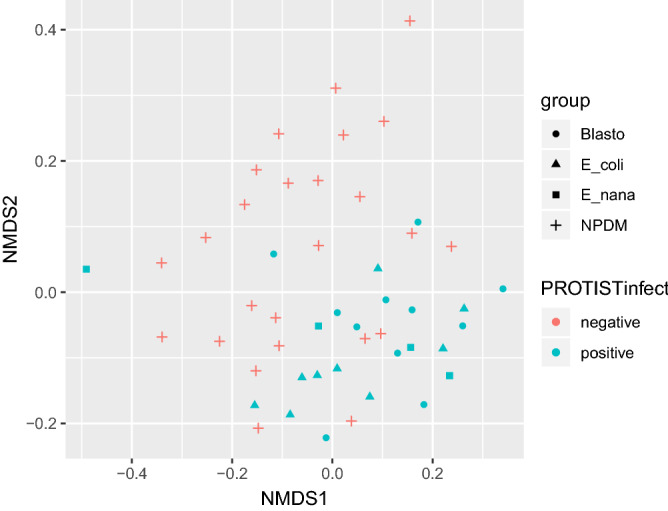
Ordination analysis of the studied children groups. Ordination analysis plot showing the calculated distances (NMDS and Bray) of the studied children in the four tested groups: **NPDM** (+ controls with no parasites detected on the microscopic stool analysis), **Blasto** ( • Children parasitized only by *Blastocystis* spp.). **E_coli** ( **∆** Children parasitized only by *Entamoeba coli*). **E_nana** (□ Children parasitized only by *Endolimax nana*)*.* Colored in red are the uninfected control children (NPDM), while in blue are the Protist infected children (colonized with either *Blastocystis spp., Entamoeba coli*, or *Endolimax nana*).

Finally, Fig. 2 shows the ordination plot using NMDS distance, which mostly separates the individuals of both groups. We determined which variables most strongly affected the structure of the children gut microbiota using a permutational multivariate analysis of variance (PERMANOVA) test of the Bray–Curtis dissimilarities. Again, the ordination analysis plot shows a significant segregation pattern (PERMANOVA R^2^ = 0.036, p-value < 0.05) of most of the non-parasites controls versus the PROTIST-infect group.

### Bacterial microbiota profiles associated with *Blastocystis spp.*, *Entamoeba. coli* or *Endolimax nana*

**Figure 3:**
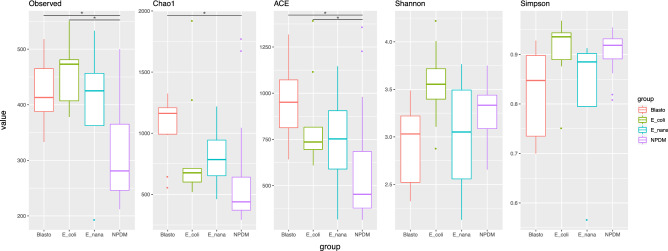
Richness and diversity indices of the control and the colonized children either with *Blastocystis* spp., *Entamoeba coli*, or *Endolimax nana*. Boxplot representation of the median values of the Observed OTUs and the indices Chao1, ACE, Shannon, and Simpson; comparing the control children (no parasites detectable on the stool analysis—**NPDM**) and the **Blasto** ( Children parasitized only by *Blastocystis* spp.), **E_coli** ( Children parasitized only by *Entamoeba coli*), and **E_nana** ( Children parasitized only by *Endolimax nana*) parasitized groups*.* The observed differences in the median values of the NPDM controls vs. Blasto or E_coli groups were statistically significant using the Wilcoxon rank-sum test, with p < 0.05 for observed OTUs and ACE index. The Chao1 index was statistically significant between NPDM controls and Blasto groups with a p < 0.01 using the same test.

The next step in our analysis was oriented to discriminate if there was any differential relationship between the specific protist harbored by each individual and the microbiota profiles. To do so, we separated the infected children into three groups regarding the detected parasite: *Blastocystis spp.*, *E. coli*, or *E. nana*; and compare them with the NPDM controls. Following the previous observations, the median values of the richness indices observed OTUs, Chao1, and ACE were augmented in all the infected groups (Fig. 3). The species richness (Observed OTUs) showed statistical significance, applying the Kruskal–Wallis rank-sum test, among all four tested groups (p-value = 0.0004175) and proved to be significantly higher in pairwise comparisons with the Wilcoxon rank-sum test between the *Blastocystis* spp. (median 413) and *E. coli* (median 473) parasitized groups (*Blasto* and E_coli, p = 0.0043 and p = 0.0010, respectively) compared to the control group (NPDM, median = 281). Additionally, the Kruskal–Wallis test showed statistical differences within the groups in all measured indices (Simpson, p-value = 0.004808; ACE, p-value = 0.002159; Chao1, p-value = 0.001095; Shannon, p-value = 0.03467 (Age-adjusted)). The Pairwise comparisons using the Wilcoxon rank-sum test indicated that the Chao1 index was only significantly different between the controls (median = 439) and the *Blasto* (median = 1,162, p = 0.0016) groups. The ACE index showed similar results in these two groups (NMPD, median 453; vs. *Blasto*, median 951; p = 0.0019). Additionally, this index showed significant differences among the NMPD controls vs. E_coli group (p = 0.0313). The Shannon and Simpson indices median values showed slight variations but were not statistically significant with the Wilcoxon rank-sum test when the controls were compared with the parasitized groups (Fig. 3).

Again, the ordination analysis plot shows a segregation pattern of most of the non-parasites controls versus the *Blastocystis spp.*, *E. coli*, or *E. nana* groups. Among the parasitized groups, it is not possible to observe an apparent clustering of the individual based on each protist species (Fig. 2).

In general, the most copious Phyla were Bacteroidetes and Firmicutes, followed by Proteobacteria, Actinobacteria, and Verrucomicrobia. This last Phylum was observed significatively more abundant in the E_coli (median = 470) group compare to the Blasto group (median = 0) (Wilcoxon rank-sum test, p = 0.019). Remarkably, two individuals of the NMPD control group presented a significant proportion of Fusobacteria (SupplementaryFigure 1). At the family taxonomic category, we observed that the taxa Prevotellaceae, Ruminococcaceae, Bacteroidaceae, and Lachnospiraceae were the most prevalent across all samples. It is noteworthy that the bars of Prevotellaceae are more prominent in the Blasto group samples compare to NPDM controls. Conversely, the control group seems to have a higher relative proportion of Ruminococcaceae compared to *Blastocystis* spp. parasitized individuals (SupplementaryFigure 2).

**Figure 4:**
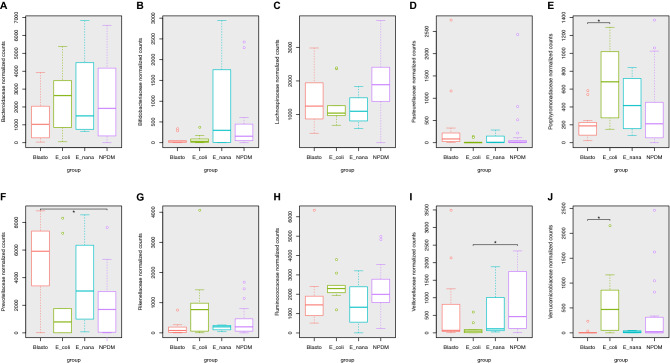
Relative abundance of the top ten most abundant bacterial families across the tested groups. Box plot graphic representation of the normalized median counts within the tested groups: **NPDM** (Controls with no parasites detected in the microscopic stool analysis), **Blasto** (Children parasitized only by *Blastocystis* spp.). **E_coli** (Children parasitized only by *Entamoeba coli*). **E_nana** (Children parasitized only by *Endolimax nana*)*.* **A** Bacteroidaceae. **B** Bifidobacteriaceae. **C** Lachnospiraceae. **D** Pasteurellaceae. **E** Porphyromonadaceae. The difference in the median values between E_coli and Blasto groups was statistically significant with a p = 0.021. **F** Prevotellaceae. The difference in the median values between NPDM controls and Blasto groups was statistically significant with a p = 0.013. **G** Rikenellaceae. **H** Ruminococcaceae. **I** Veillonellaceae. The difference in the median values between NPDM controls and E_coli groups was statistically significant with a p = 0.031. **J** Verrucomicrobiaceae. The difference in the median values between E_coli and Blasto groups was statistically significant with a p = 0.019.

Quantitative comparisons of the relative abundance of the top 10 most abundant classified bacterial families showed significative (Kruskal–Wallis test) changes in Prevotellaceae (p-value = 0.01638), Porphyromonadaceae (p-value = 0.03995), Veillonellaceae (p-value = 0.04452), Verrucomicrobiaceae (p-value = 0.0165), and Pasteurellaceae (p-value = 0.04312) (Fig. 4).

Prevotellaceae and Verrucomicrobiaceae also showed the best statistical significance in the pairwise comparisons of the relative abundances using the Wilcoxon rank-sum test. For Prevotellaceae, the significative difference (p-value = 0.01638) was observed between the Blasto (median = 5,912) and the NMPD controls (1696). In the case of Verrucomicrobiaceae, median values drop from 470 in the E_coli group to 0 in the NPDM controls (p-value = 0.019). Porphyromonadaceae also showed significant results between the Blasto and E_coli groups (p-value = 0.021), while in Veillonellaceae the significative differences were observed between the NPDM controls and the E_coli group (p-value = 0.031).

**Figure 5:**
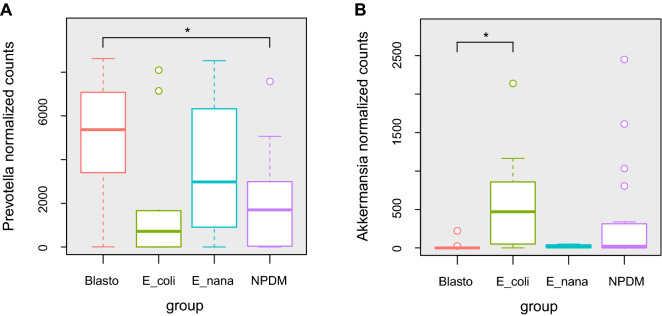
A. Relative abundance of Prevotella across tested groups. Box plot graphic representation of the normalized median counts of Prevotella within the tested groups: **NPDM** (Controls with no parasites detected in the microscopic stool analysis), **Blasto** (Children parasitized only by *Blastocystis* spp.). **E_coli** (Children parasitized only by *Entamoeba coli*). **E_nana** (Children parasitized only by *Endolimax nana*)*.* The difference in the median values between NPDM controls and Blasto groups was statistically significant with a p < 0.05. **C. Relative abundance of Akkermansia across tested groups.** Box plot graphic representation of the normalized median counts of Akkermansia within the tested groups: **NPDM** (Controls with no parasites detected in the microscopic stool analysis), **Blasto** (Children parasitized only by *Blastocystis* spp.). **E_coli** (Children parasitized only by *Entamoeba coli*). **E_nana** (Children parasitized only by *Endolimax nana*)*.* The difference in the median values between Blasto and E_coli groups was statistically significant with a p < 0.05.

At the genus category, *Prevotella*, *Bacteroides*, and *Faecalibacterium* were, in order, the dominant microoganisms over all individuals (SupplementaryFigure 3). These results indicate changes in *Prevotella* proportions, which seem to be enriched in the Blasto group. One more detailed and quantitative analysis on *Prevotella* showed an increase in the median values of normalized counts of this anaerobe in the Blasto (5,367) group compared to the controls (1696) that were statistically significative (p = 0.011) (Fig. 5A).

### *Prevotella* is enriched within the individuals harboring *Blastocystis *while *Akkermansia *seems to be favored with *Entamoeba coli* colonization

To further confirm the previous findings on the changes in the relative abundance of the mentioned microbe families, a LEfSe analysis was performed, aiming to identify marker microorganisms within the tested groups. After filtering the results excluding the unclassified organisms and LDA score above 3, 15 OTUs were kept with significant p values (Table 1). *Prevotella* (OTU00001) showed the highest abundance shift with an LDA score of 5.32 towards the Blasto group. This group was also enriched with the bacteria *Haemophilus*, *Holdemanella*, and *Butyricicoccus*.

**Table 1:** LEfSe analyses of the marker microorganisms of each tested group.

OTU	LogMaxMean	Class	LDA	pValue	OTUsize	Phylum	Family	Genus
Otu00015	4.11044	NPDM	3.75427	0.0195828	10,821	Firmicutes	Lachnospiraceae(100)	Blautia(100)
Otu00085	3.50502	NPDM	3.47811	0.01566	1924	Firmicutes	Streptococcaceae(100)	Streptococcus(100)
Otu00122	3.576	E_nana	3.16078	0.00075462	1,020	Bacteroidetes	Prevotellaceae(100)	Paraprevotella(100)
Otu00005	4.67852	E_coli	4.56941	0.0158793	21,936	Verrucomicrobia	Verrucomicrobiaceae(100)	Akkermansia(100)
Otu00075	3.71921	E_coli	3.47399	0.0314539	2,442	Firmicutes	Lachnospiraceae(100)	Coprococcus(100)
Otu00141	3.36365	E_coli	3.06432	0.0130117	741	Bacteroidetes	Rikenellaceae(100)	Alistipes(100)
Otu00001	5.48881	blasto	5.32462	0.0324499	181,806	Bacteroidetes	Prevotellaceae(100)	Prevotella(100)
Otu00010	4.51725	blasto	4.43657	0.0386207	15,792	Proteobacteria	Pasteurellaceae(100)	Haemophilus(100)
Otu00068	3.60055	blasto	3.42977	0.0354466	2,802	Firmicutes	Erysipelotrichaceae(100)	Holdemanella(100)
Otu00088	3.4176	blasto	3.3222	0.0298255	1836	Firmicutes	Ruminococcaceae(100)	Butyricicoccus(100)

The second highest score was obtained for *Akkermansia* (LDA 4.57) in the E_coli group, which was also enriched with *Coprococcus* and *Alistipes*. The control group had two enriched biomarker organisms, both from the Firmicutes phylum, *Blautia*, and *Streptococcus*. The case of *Akkermansia* is quite impressive since it is not included in the top ten most abundant organisms, although it was the second most abundant biased OTU in the tested groups. To gain insights into this particular group, the normalized counts of *Akkermansia* were extracted and plotted (Fig. 5B). These results display a reduced number of counts in the Blasto group with a median value of zero, while the control and the E_coli group, 23 and 470, respectively. The Kruskal–Wallis test showed statistical significance in the differences among groups (p-value = 0.0165), and the Pairwise Wilcoxon test showed there only significant differences between the Blasto and the E_coli groups (p = 0.019).

## Discussion

Children distal intestinal microbiota tends to stabilize and begin to be more adult-like around 3-yo^8^. In our selected children group only two were younger than 2-yo, most are in the range of 3 to 5-yo, when intestinal microbiota starts to stabilize. This can explain why, albeit the described association of gut microbiota diversity and age in children, a weak correlation with the Shannon diversity index was observed. Additionally, since these children receive most of their meals in the daycare center, allowing them to have a more similar intestinal microbiota due to the effect of a normalized diet. Nonetheless, despite the influence of age within the intestinal microbiota in the studied children, our results showed that Protist colonization have a relevant impact on the intestinal microbial community in children.

Eukaryotic parasites are major competitors in the microbial world due to bacterivorous activity or direct competition for nutrients^27,53,54^. The effect of intestinal nematodes and protists on the human gut bacterial microbiota has been proved in several studies, and some of them show, as a common trend, that bacterial richness is increased in individuals that carry intestinal parasites. However, diversity is not always augmented as well^20–23,28–30,52^.

In the present study, we observed that all the studied protist, *Blastocystis spp.*, *Entamoeba coli,* and *Endolimax nana;* were associated with a significant increase in bacterial richness, with median values that almost doubles the control group. We cannot conclude if this is cause or effect, but these findings are in concordance with previous reports for similar studies on the relation of *Blastocystis* spp. with the intestinal microbiota. For *Entamoeba coli* and *Endolimax nana* it was not possible to find previous scientific publications addressing this topic. To the best of our knowledge, this is the first report that observes the relation of the intestinal gut microbiota and these two amoebae using a 16S metataxonomic approach. It is essential to highlight that no definitive conclusion can be drawn from the individuals infected with *Endolimax nana* due to the low number of children included in this group, only 4. *Endolimax nana* is an intestinal amoeba of humans that has a cosmopolitan distribution, most likely as a commensal or nonpathogenic protozoon, with an estimated global prevalence in healthy individuals of 13.4%. The scientific evidence to date is inconclusive in terms of its host specificity, epidemiology, morphology, taxonomy and genetic diversity^55^. Although some authors suggest that *E. nana* feed exclusively on bacteria and that it could have a pathogenic potential, with case reports of parasitized patients suffering arthritis^56^, intestinal symptoms^57–59^, and urticaria^60,61^, there is not enough evidence that supports this statements. Therefore, studies focused on the parasite genetic variability and crosstalk interaction with the microbiota and the immune system are needed to provide data that clarify the effects of this protozoan in the human intestine. Furthermore, the fecal–oral transmission suggests that *Endolimax* can be used as a biological marker suitable for the hygiene measures of the population and fecal contamination of food or water^62^.

*Blastocystis *spp*.* has been associated in several studies with increased bacterial diversity in western European adults and Mexicans^30,46^. In our study in Colombian children, although we found an increased richness in the colonized individuals, no significant differences were observed in the diversity indices. This controversial finding might be attributed to a differential response in the child or adult intestinal microbiota to the *Blastocystis* challenge. More studies in children need to be performed in order to understand in detail this phenomenon.

Since its first observation in 1849, *Blastocystis* spp*.* was initially described as a pathogenic parasite being formally termed in early 20th Century^63,64^. After several decades of debate about its classification and host preferences, in the second half of this century, it was generally accepted as a pathogenic protist for humans that can cause diarrhea and abdominal pain^65^. However, several researchers have raised concerns about the evidence that supports the pathogenicity of *Blastocystis* spp. in humans, and its clinical significance remains controversial^66–68^. In vitro and in vivo studies demonstrate pathogenic potential but also show considerable inter and intra subtype variation, which provides a possible explanation on the conflicting reports on clinical significance. *Blastocystis* spp. have intestinal immunomodulatory effects and can release proteases that affect the integrity of the epithelium. This situation might facilitate colonization by other enteric pathogens either directly or by the resultant changes in the gut microbiota^69^.

Our findings unveil that children carrying *Blastocystis* spp. display a different microbial community compared to uninfected controls with a tendency to a Prevotella-driven enterotype. This is in concordance with similar studies carried out in adults around the world^28–30^. *Blastocystis* spp. showed a significant shift in the *Prevotella* proportion enriching it, favoring an enterotype switch. *Prevotella* strains are associated with plant-rich diets, including fibers, simple sugars, and carbohydrates, suggesting that it is a beneficial microbe^70^. However, *Prevotella* in the gut has also been linked with inflammatory diseases, which made it difficult to predict its behavior in any given gut ecosystem^70^. Our results are similar to those found in healthy children from several developing countries, who had a gut microbiome dominated at the genus rank by *Prevotella*^71–73^. High species diversity at this genus could be related to its different effects on host health.

Andersen et al. found that *Blastocystis* spp. colonization was positively associated with species richness, and this parameter was negatively correlated with the *Bacteroides*-driven enterotype. The authors concluded that *Blastocystis* spp. establishment in the intestine probably depends on the activity of certain types of bacteria that are generally not present in individuals with low richness colonic microbiota^74^. Since *Blastocystis* spp. is an obligated anaerobe, in order to survive, it should favor the predominance of bacterial taxa that maintains a strict anaerobic environment in the gut lumen^75^.

An interesting finding was that *Akkermansia*, a bacterium effective in increasing mucus thickness and gut barrier function, was reduced in the *Blastocystis* spp. infected group, suggesting that this protist poses an unfavorable condition to this beneficial bacterium. This reduction has been previously described, and it was related to specific *Blastocystis* subtypes, with an inverse correlation of subtypes 3 and 4 with *Akkermansia*, suggesting differential associations between subtypes and host health^29^. Other authors have also shown that *Blastocystis* spp. infection also leads to changes in the abundance of other groups of bacteria, reducing *Bacteroides*, and increasing *Prevotella*^28,29,75^.

*Entamoeba coli* colonized children showed a bacterial community that closely resembles the control group without a Prevotella-driven enterotype. We also found an increase in the relative abundance of the beneficial bacterium *Akkermansia* in this group*,* contrary to the pattern observed in the *Blastocystis* spp. infected children. This commensal amoeba probably contributes to maintaining gut favorable conditions to beneficial bacteria, like *Akkermansia*. Although it is challenging to fully interpret the role of any microorganism in a complex community such as the gut microbiota, our results indicate that commensal protists like *Entamoeba coli* could be related to a healthy status.

Nowadays, the definition of the pathogenicity of intestinal parasites should not only be restricted to its capacity to alter or invade the intestinal mucosa, but the alteration of the healthy gut microbiota might also be a cause of disease^1,14,76,77^. The alteration profile of the distal microbiota observed in the individuals colonized by *Blastocystis* spp. have been associated with intestinal bowel disease, and a reduced abundance of *Akkermansia* is associated with diseases like Atherosclerosis^78^, ulcerative colitis^79^, appendicitis^80^, overweight and obesity^81^. From this point of view, changes in the intestinal gut microbiota seem to correlate or exacerbate several diseases, so it should be considered at the moment of defining the pathogenic capacity of a parasite. It seems clear with the actual scientific evidence that *Blastocystis* spp. has the power to promote the displacement of the “healthy intestinal microbiota”, rendering the children more susceptible to other diseases thanks to the increase in *Prevotella* and the reduction of *Akkermansia*. By definition, a commensal parasite, like *Entamoeba coli*, should not affect the normal physiology of the host. However, the evidence shown in this paper add arguments in favor of the pathogenic behavior of *Blastocystis* spp. in children.

## Methods

### Population and sample selection

Children attending seven public daycare centers in the three nearby neighborhoods in Medellin, Colombia, were selected for this study. Feces samples were collected in screw-capped containers without any preservatives and then transported to the lab within a maximum period of 3 h. Samples for DNA extraction were frozen at − 70 °C for a period that did not exceed 7 days, and then DNA was extracted. The microscopical parasitological analysis was performed on the same day of the collection by observation of direct (fresh and iodine solution) and modified Ritchie concentrated stool samples. Modified Ziehl–Neelsen slides of the feces samples were also prepared and observed microscopically to detect intestinal apicomplexan parasites. Specific PCR for *Cryptosporidium* was performed using the protocol described by Xiao et al*.*^82,83^*.* All selected samples were negative with the test mentioned above for intestinal apicomplexan parasites.

Subjects were selected based on being positive for any of the following protists: *Blastocystis* spp., *Entamoeba coli*, or *Endolimax nana*. A control group negative for intestinal parasites was also included. These children received the same food (breakfast and lunch) while assisting the daycare centers and the age ranged from one to five years old (1yo, n = 2; 2yo, n = 11; 3yo, n = 16, 4yo, n = 9, 5yo, n = 11). By sex, the distribution was 17 females and 32 males. Enrolled children were classified into four groups:

**NPDM**: Control group of children with no positive results for parasites (n = 25). **Blasto**: Children parasitized only by *Blastocystis* spp., no other parasites observed (n = 11). **E_coli**: Children parasitized only by *Entamoeba coli*, no other parasites observed (n = 9). **E_nana**: Children parasitized only by *Endolimax nana*, no other parasites observed (n = 4)*.* The **Protist-infect** group was set adding all the children of the Blasto, E_coli and E_nana groups (n = 24).

### DNA extraction and 16S metataxonomic experiment

DNA extraction was carried with the STOOL DNA kit NORGEN (Canada). Extracted DNA was quantified using UV absorption and the Picogreen fluorescent method. The DNA quality was assessed by gel electrophoresis and control PCR amplifying the complete eDNA 16S gene with universal primers 27F and 1492R. For the metataxonomic experiment, the primers Bakt_341F CCTACGGGNGGCWGCAG and Bakt_805R GACTACHVGGGTATCTAATCC, that amplify the V3/V4 region, were used. The 16S metataxonomic experiment was hired to MACROGEN, Korea. An average of 100.000 reads per library was generated in the MiSeq platform with PE reads of 300 bases. For further bioinformatic analysis 50,000 reads were randomly selected for each sample with the program SEQTK (https://github.com/lh3/seqtk).

### Bioinformatic analysis

Amplicon processing was carried out with MOTHUR V1.42^84^. Following the MiSeq standard operating protocol provided by the authors (https://www.mothur.org/wiki/MiSeq_SOP). Briefly, Amplicon forward and reverse read were merged and those containing Ns or homopolymers larger than 6 bases were removed. Then, the reads were mapped to the SILVA database, and only those that mapped to the V3/V4 region were kept. Chimera removal was performed with VSEARCH^85^. Reads per library were rarified with the *totalgroup* strategy to an average of 12,249 clean sequences. OTUs supported by less than 4 reads were excluded. A BIOM file was prepared with the MOTHUR function make.biom, for further statistical analysis in R language.

### Statistics analysis

The statistical analysis was performed with package PHYLOSEQ in the R environment. The data was imported as a BIOM file generated with MOTHUR. Alpha diversity statistics were calculated and plotted in boxplots. The ordination plot was generated with NMDS and BRAY distances. Read counts per library were normalized with median sequencing depth. With these normalized counts, we compared taxa abundance at Phylum, family, and genus categories and generated the bar plots of the top ten most abundant taxa, the remaining groups were gathered in the others category. Statistical significance of the differences in the median values was performed with Kruskal–Wallis and pairwise Wilcoxon rank-sum test. The age effect on the Shannon index was assessed using the ‘lm’ function. It was adjusted by subtracting the age contribution to the index.

Permutational multivariate analysis of variance (PERMANOVA) was performed using the Adonis function in the vegan library of R with the Bray–Curtis dissimilarity matrix.

### Ethics statement

The ethical clearance of this study was followed by the ethics of the Helsinki declaration and resolution No. 008430 of 1993 from the Ministry of Health from Colombia. The study was approved by the Ethics Committee from Sede de Investigación Universitaria, Universidad de Antioquia, under the official document No. 14-06-564. Parents or legal guardians of all the enrolled individuals signed informed consent.

## Supplementary information

Supplementary information

## Data Availability

Raw data is available upon publication at SRA website bioproject PRJNA572583. The datasets generated during and/or analyzed during the current study are also available from the corresponding author on reasonable request.
