# Supplementary material for: Contrasting microbiota profiles observed in children carrying either Blastocystis spp. or the commensal amoebas Entamoeba coli or Endolimax nana
Source: Sci Rep. 2020 Sep 18;10:15354. doi: 10.1038/s41598-020-72286-y (PMC7501860; doi:10.1038/s41598-020-72286-y)
Supplement: Supplementary file 1 — Supplementary information [file 41598_2020_72286_MOESM1_ESM.docx]

**Contrasting microbiota profiles observed in children carrying either *Blastocystis spp.* or the commensal amoebas *Entamoeba coli* or *Endolimax nana***

Juan F. Alzate^1,2,*^, Miguel Toro-Londoño^2^, Felipe Cabarcas^2,3^, Gisela Garcia-Montoya^1^ and Ana Galvan-Diaz ^4^.

^1^ Departamento de Microbiología y Parasitología, Facultad de Medicina, Universidad de Antioquia, Medellín, Colombia

^2^ Centro Nacional de Secuenciación genómica – CNSG, Sede de Investigación Universitaria – SIU, Facultad de Medicina, Universidad de Antioquia

^3^ Grupo Sistemic, Facultad de Ingeniería, Universidad de Antioquia, Medellín, Colombia

^4^ Grupo de Microbiología ambiental, Escuela de Microbiología, Universidad de Antioquia, Medellín, Colombia

^*^ Corresponding Author: jfernando.alzate@udea.edu.co

**Supplementary results**

SR1. Results of the Kruskal-Wallis rank sum test for diversity indices (Shannon and Simpson) vs Sex and Age:

Kruskal-Wallis rank sum test SEX

*data: rich$Shannon and sample_data(myData_CNSG)$Sex*

Kruskal-Wallis chi-squared = 1.1029, df = 1, p-value = 0.2936

*data: rich$Simpson and sample_data(myData_CNSG)$Sex*

Kruskal-Wallis chi-squared = 1.0593, df = 1, p-value = 0.3034

Kruskal-Wallis rank sum test AGE

*data: rich$Shannon and sample_data(myData_CNSG)$Age*

Kruskal-Wallis chi-squared = 7.1621, df = 4, p-value = 0.1276

*data: rich$Simpson and sample_data(myData_CNSG)$Age*

Kruskal-Wallis chi-squared = 4.6556, df = 4, p-value = 0.3245

Spearman correlation (diversity metrics vs age)

Spearman's rank correlation rho

data: indices_data$Age and indices_data$value (Simpson)

S = 19422, p-value = 0.9507

alternative hypothesis: true rho is not equal to 0

sample estimates:

rho 0.009067256

Spearman's rank correlation rho

data: indices_data$Age and indices_data$value (Shannon)

S = 13334, p-value = 0.02515

alternative hypothesis: true rho is not equal to 0

sample estimates:

rho 0.3196735

**Age-Shannon lm model statistics**

Residuals:

Min 1Q Median 3Q Max

-1.25950 -0.27889 0.07902 0.26376 0.93438

Coefficients:

Estimate Std. Error t value Pr(>|t|)

(Intercept) 2.88690 0.18264 15.806 <2e-16 ***

Age 0.10003 0.05181 1.931 0.0596 .

---

Signif. codes: 0 ‘***’ 0.001 ‘**’ 0.01 ‘*’ 0.05 ‘.’ 0.1 ‘ ’ 1

Residual standard error: 0.4234 on 47 degrees of freedom

Multiple R-squared: 0.07349, Adjusted R-squared: 0.05377

F-statistic: 3.728 on 1 and 47 DF, p-value: 0.05956

SR2. Pairwise comparisons using Wilcoxon rank sum test among all the tested groups; parasitized and controls.

data: rich$Observed and sample_data(myData_CNSG)$group

Blasto E_coli E_nana

E_coli 0.7334 - -

E_nana 1.0000 1.0000 -

NPDM 0.0043 0.0010 0.7589

P value adjustment method: holm

data: rich$Shannon and sample_data(myData_CNSG)$group

Blasto E_coli E_nana

E_coli **0.0099** - -

E_nana 1.0000 0.7804 -

NPDM 0.0655 0.1804 1.0000

P value adjustment method: holm

data: rich$Chao1 and sample_data(myData_CNSG)$group

Blasto E_coli E_nana

E_coli 0.4558 - -

E_nana 0.4558 0.7105 -

NPDM 0.0016 0.0528 0.4352

P value adjustment method: holm

data: rich$ACE and sample_data(myData_CNSG)$group

Blasto E_coli E_nana

E_coli 0.9195 - -

E_nana 1.0000 1.0000 -

NPDM 0.0019 0.0313 1.0000

P value adjustment method: holm

data: rich$Simpson and sample_data(myData_CNSG)$group

Blasto E_coli E_nana

E_coli **0.048** - -

E_nana 0.949 0.328 -

NPDM 0.010 0.560 0.328

P value adjustment method: holm

SR3. Quantitative comparisons of the relative abundance of the top 10 most abundant bacterial families.

**BacteroidaceaeCount**

> group_by(PhylumFamilyGenusCounts, group) %>% summarise(count = n(), mean = mean(BacteroidaceaeCount, na.rm = TRUE), sd = sd(BacteroidaceaeCount, na.rm = TRUE), median = median(BacteroidaceaeCount, na.rm = TRUE),IQR = IQR(BacteroidaceaeCount, na.rm = TRUE))

Object of class "tbl_dftbldata.frame"

group count mean sd median IQR

1 Blasto 11 1249.000 1192.676 1032.0 1765.5

2 E_coli 9 2430.222 1896.872 2638.0 2621.0

3 E_nana 4 2616.000 2881.656 1502.5 2503.0

4 NPDM 25 2356.920 2057.750 1926.0 3791.0

> kruskal.test(sample_data(myData_CNSG)$BacteroidaceaeCount, sample_data(myData_CNSG)$group, pvalue.threshold = 0.05)

Kruskal-Wallis rank sum test

data: sample_data(myData_CNSG)$BacteroidaceaeCount and sample_data(myData_CNSG)$group

Kruskal-Wallis chi-squared = 2.6211, df = 3, p-value = 0.4538

> pairwise.wilcox.test(sample_data(myData_CNSG)$BacteroidaceaeCount, sample_data(myData_CNSG)$group)

Pairwise comparisons using Wilcoxon rank sum test

data: sample_data(myData_CNSG)$BacteroidaceaeCount and sample_data(myData_CNSG)$group

Blasto E_coli E_nana

E_coli 1 - -

E_nana 1 1 -

NPDM 1 1 1

P value adjustment method: holm

**RuminococcaceaeCount**

> group_by(PhylumFamilyGenusCounts, group) %>% summarise(count = n(), mean = mean(RuminococcaceaeCount, na.rm = TRUE), sd = sd(RuminococcaceaeCount, na.rm = TRUE), median = median(RuminococcaceaeCount, na.rm = TRUE),IQR = IQR(RuminococcaceaeCount, na.rm = TRUE))

Object of class "tbl_dftbldata.frame"

group count mean sd median IQR

1 Blasto 11 1802.364 1605.6373 1449.0 996

2 E_coli 9 2372.333 724.3658 2293.0 373

3 E_nana 4 1470.000 1329.9421 1331.5 1136

4 NPDM 25 2192.600 1113.8073 1997.0 1202

> kruskal.test(sample_data(myData_CNSG)$RuminococcaceaeCount, sample_data(myData_CNSG)$group, pvalue.threshold = 0.05)

Kruskal-Wallis rank sum test

data: sample_data(myData_CNSG)$RuminococcaceaeCount and sample_data(myData_CNSG)$group

Kruskal-Wallis chi-squared = 6.2445, df = 3, p-value = 0.1003

> pairwise.wilcox.test(sample_data(myData_CNSG)$RuminococcaceaeCount, sample_data(myData_CNSG)$group)

Pairwise comparisons using Wilcoxon rank sum test

data: sample_data(myData_CNSG)$RuminococcaceaeCount and sample_data(myData_CNSG)$group

Blasto E_coli E_nana

E_coli 0.12 - -

E_nana 0.85 0.79 -

NPDM 0.50 0.84 0.84

P value adjustment method: holm

**LachnospiraceaeCount**

> group_by(PhylumFamilyGenusCounts, group) %>% summarise(count = n(), mean = mean(LachnospiraceaeCount, na.rm = TRUE), sd = sd(LachnospiraceaeCount, na.rm = TRUE), median = median(LachnospiraceaeCount, na.rm = TRUE),IQR = IQR(LachnospiraceaeCount, na.rm = TRUE))

Object of class "tbl_dftbldata.frame"

group count mean sd median IQR

1 Blasto 11 1476.273 792.0445 1249.0 1076.50

2 E_coli 9 1317.333 612.6137 1042.0 296.00

3 E_nana 4 1152.250 518.6369 1095.5 407.25

4 NPDM 25 1881.520 895.5733 1886.0 1020.00

> kruskal.test(sample_data(myData_CNSG)$LachnospiraceaeCount, sample_data(myData_CNSG)$group, pvalue.threshold = 0.05)

Kruskal-Wallis rank sum test

data: sample_data(myData_CNSG)$LachnospiraceaeCount and sample_data(myData_CNSG)$group

Kruskal-Wallis chi-squared = 6.2048, df = 3, p-value = 0.1021

> pairwise.wilcox.test(sample_data(myData_CNSG)$LachnospiraceaeCount, sample_data(myData_CNSG)$group)

Pairwise comparisons using Wilcoxon rank sum test

data: sample_data(myData_CNSG)$LachnospiraceaeCount and sample_data(myData_CNSG)$group

Blasto E_coli E_nana

E_coli 1.00 - -

E_nana 1.00 1.00 -

NPDM 0.73 0.24 0.47

P value adjustment method: holm

**BifidobacteriaceaeCount**

> group_by(PhylumFamilyGenusCounts, group) %>% summarise(count = n(), mean = mean(BifidobacteriaceaeCount, na.rm = TRUE), sd = sd(BifidobacteriaceaeCount, na.rm = TRUE), median = median(BifidobacteriaceaeCount, na.rm = TRUE),IQR = IQR(BifidobacteriaceaeCount, na.rm = TRUE))

Object of class "tbl_dftbldata.frame"

group count mean sd median IQR

1 Blasto 11 75.00000 111.1476 44.0 38.0

2 E_coli 9 87.22222 116.3032 35.0 81.0

3 E_nana 4 884.00000 1394.9946 299.5 1156.5

4 NPDM 25 387.64000 622.3042 157.0 399.0

> kruskal.test(sample_data(myData_CNSG)$BifidobacteriaceaeCount, sample_data(myData_CNSG)$group, pvalue.threshold = 0.05)

Kruskal-Wallis rank sum test

data: sample_data(myData_CNSG)$BifidobacteriaceaeCount and sample_data(myData_CNSG)$group

Kruskal-Wallis chi-squared = 6.5317, df = 3, p-value = 0.08842

> pairwise.wilcox.test(sample_data(myData_CNSG)$BifidobacteriaceaeCount, sample_data(myData_CNSG)$group)

Pairwise comparisons using Wilcoxon rank sum test

data: sample_data(myData_CNSG)$BifidobacteriaceaeCount and sample_data(myData_CNSG)$group

Blasto E_coli E_nana

E_coli 1.00 - -

E_nana 1.00 1.00 -

NPDM 0.12 0.32 1.00

P value adjustment method: holm

Warning messages:

1: In wilcox.test.default(xi, xj, paired = paired, ...) :

cannot compute exact p-value with ties

**PorphyromonadaceaeCount**

> group_by(PhylumFamilyGenusCounts, group) %>% summarise(count = n(), mean = mean(PorphyromonadaceaeCount, na.rm = TRUE), sd = sd(PorphyromonadaceaeCount, na.rm = TRUE), median = median(PorphyromonadaceaeCount, na.rm = TRUE),IQR = IQR(PorphyromonadaceaeCount, na.rm = TRUE))

Object of class "tbl_dftbldata.frame"

group count mean sd median IQR

1 Blasto 11 213.7273 181.4443 188 141.50

2 E_coli 9 703.5556 413.3519 681 740.00

3 E_nana 4 437.7500 343.9529 415 459.75

4 NPDM 25 368.4800 385.4942 212 397.00

> kruskal.test(sample_data(myData_CNSG)$PorphyromonadaceaeCount, sample_data(myData_CNSG)$group, pvalue.threshold = 0.05)

Kruskal-Wallis rank sum test

data: sample_data(myData_CNSG)$PorphyromonadaceaeCount and sample_data(myData_CNSG)$group

Kruskal-Wallis chi-squared = 8.3139, df = 3, **p-value = 0.03995**

> pairwise.wilcox.test(sample_data(myData_CNSG)$PorphyromonadaceaeCount, sample_data(myData_CNSG)$group)

Pairwise comparisons using Wilcoxon rank sum test

data: sample_data(myData_CNSG)$PorphyromonadaceaeCount and sample_data(myData_CNSG)$group

Blasto E_coli E_nana

E_coli **0.021** - -

E_nana 0.903 0.903 -

NPDM 0.984 0.175 0.984

P value adjustment method: holm

Warning messages:

1: In wilcox.test.default(xi, xj, paired = paired, ...) :

cannot compute exact p-value with ties

**RikenellaceaeCount**

> group_by(PhylumFamilyGenusCounts, group) %>% summarise(count = n(), mean = mean(RikenellaceaeCount, na.rm = TRUE), sd = sd(RikenellaceaeCount, na.rm = TRUE), median = median(RikenellaceaeCount, na.rm = TRUE),IQR = IQR(RikenellaceaeCount, na.rm = TRUE))

Object of class "tbl_dftbldata.frame"

group count mean sd median IQR

1 Blasto 11 148.7273 215.12838 78 177.0

2 E_coli 9 969.4444 1250.68912 769 926.0

3 E_nana 4 173.5000 98.91916 198 106.5

4 NPDM 25 375.0800 450.68161 198 407.0

> kruskal.test(sample_data(myData_CNSG)$RikenellaceaeCount, sample_data(myData_CNSG)$group, pvalue.threshold = 0.05)

Kruskal-Wallis rank sum test

data: sample_data(myData_CNSG)$RikenellaceaeCount and sample_data(myData_CNSG)$group

Kruskal-Wallis chi-squared = 5.5055, df = 3, p-value = 0.1383

> pairwise.wilcox.test(sample_data(myData_CNSG)$RikenellaceaeCount, sample_data(myData_CNSG)$group)

Pairwise comparisons using Wilcoxon rank sum test

data: sample_data(myData_CNSG)$RikenellaceaeCount and sample_data(myData_CNSG)$group

Blasto E_coli E_nana

E_coli 0.28 - -

E_nana 0.99 0.99 -

NPDM 0.63 0.69 0.99

P value adjustment method: holm

Warning messages:

1: In wilcox.test.default(xi, xj, paired = paired, ...) :

cannot compute exact p-value with ties

**VeillonellaceaeCount**

> group_by(PhylumFamilyGenusCounts, group) %>% summarise(count = n(), mean = mean(VeillonellaceaeCount, na.rm = TRUE), sd = sd(VeillonellaceaeCount, na.rm = TRUE), median = median(VeillonellaceaeCount, na.rm = TRUE),IQR = IQR(VeillonellaceaeCount, na.rm = TRUE))

Object of class "tbl_dftbldata.frame"

group count mean sd median IQR

1 Blasto 11 700.6364 1137.9312 74 771.50

2 E_coli 9 122.7778 190.0130 51 90.00

3 E_nana 4 535.7500 899.2443 116 490.75

4 NPDM 25 766.7600 790.7422 464 1619.00

> kruskal.test(sample_data(myData_CNSG)$VeillonellaceaeCount, sample_data(myData_CNSG)$group, pvalue.threshold = 0.05)

Kruskal-Wallis rank sum test

data: sample_data(myData_CNSG)$VeillonellaceaeCount and sample_data(myData_CNSG)$group

Kruskal-Wallis chi-squared = 8.0733, df = 3, **p-value = 0.04452**

> pairwise.wilcox.test(sample_data(myData_CNSG)$VeillonellaceaeCount, sample_data(myData_CNSG)$group)

Pairwise comparisons using Wilcoxon rank sum test

data: sample_data(myData_CNSG)$VeillonellaceaeCount and sample_data(myData_CNSG)$group

Blasto E_coli E_nana

E_coli 0.993 - -

E_nana 0.993 0.993 -

NPDM 0.993 **0.031** 0.993

P value adjustment method: holm

Warning message:

In wilcox.test.default(xi, xj, paired = paired, ...) :

cannot compute exact p-value with ties

**VerrucomicrobiaceaeCount**

> group_by(PhylumFamilyGenusCounts, group) %>% summarise(count = n(), mean = mean(VerrucomicrobiaceaeCount, na.rm = TRUE), sd = sd(VerrucomicrobiaceaeCount, na.rm = TRUE), median = median(VerrucomicrobiaceaeCount, na.rm = TRUE),IQR = IQR(VerrucomicrobiaceaeCount, na.rm = TRUE))

Object of class "tbl_dftbldata.frame"

group count mean sd median IQR

1 Blasto 11 24.18182 65.68534 0 8.50

2 E_coli 9 619.11111 698.90029 470 807.00

3 E_nana 4 21.25000 24.25387 17 33.75

4 NPDM 25 312.52000 591.57474 23 314.00

> kruskal.test(sample_data(myData_CNSG)$VerrucomicrobiaceaeCount, sample_data(myData_CNSG)$group, pvalue.threshold = 0.05)

Kruskal-Wallis rank sum test

data: sample_data(myData_CNSG)$VerrucomicrobiaceaeCount and sample_data(myData_CNSG)$group

Kruskal-Wallis chi-squared = 10.257, df = 3, **p-value = 0.0165**

> pairwise.wilcox.test(sample_data(myData_CNSG)$VerrucomicrobiaceaeCount, sample_data(myData_CNSG)$group)

Pairwise comparisons using Wilcoxon rank sum test

data: sample_data(myData_CNSG)$VerrucomicrobiaceaeCount and sample_data(myData_CNSG)$group

Blasto E_coli E_nana

E_coli **0.019** - -

E_nana 0.742 0.301 -

NPDM 0.215 0.309 0.742

P value adjustment method: holm

Warning messages:

1: In wilcox.test.default(xi, xj, paired = paired, ...) :

cannot compute exact p-value with ties

**PasteurellaceaeCount**

> group_by(PhylumFamilyGenusCounts, group) %>% summarise(count = n(), mean = mean(PasteurellaceaeCount, na.rm = TRUE), sd = sd(PasteurellaceaeCount, na.rm = TRUE), median = median(PasteurellaceaeCount, na.rm = TRUE),IQR = IQR(PasteurellaceaeCount, na.rm = TRUE))

Object of class "tbl_dftbldata.frame"

group count mean sd median IQR

1 Blasto 11 420.09091 838.10721 85 192.0

2 E_coli 9 27.66667 49.69406 2 11.0

3 E_nana 4 76.50000 139.75574 10 74.5

4 NPDM 25 174.48000 500.98221 16 43.0

> kruskal.test(sample_data(myData_CNSG)$PasteurellaceaeCount, sample_data(myData_CNSG)$group, pvalue.threshold = 0.05)

Kruskal-Wallis rank sum test

data: sample_data(myData_CNSG)$PasteurellaceaeCount and sample_data(myData_CNSG)$group

Kruskal-Wallis chi-squared = 8.1445, df = 3, **p-value = 0.04312**

> pairwise.wilcox.test(sample_data(myData_CNSG)$PasteurellaceaeCount, sample_data(myData_CNSG)$group)

Pairwise comparisons using Wilcoxon rank sum test

data: sample_data(myData_CNSG)$PasteurellaceaeCount and sample_data(myData_CNSG)$group

Blasto E_coli E_nana

E_coli 0.089 - -

E_nana 0.508 1.000 -

NPDM 0.241 0.508 1.000

P value adjustment method: holm

Warning messages:

1: In wilcox.test.default(xi, xj, paired = paired, ...) :

cannot compute exact p-value with ties

**PrevotellaceaeCount**

> group_by(PhylumFamilyGenusCounts, group) %>% summarise(count = n(), mean = mean(PrevotellaceaeCount, na.rm = TRUE), sd = sd(PrevotellaceaeCount, na.rm = TRUE), median = median(PrevotellaceaeCount, na.rm = TRUE),IQR = IQR(PrevotellaceaeCount, na.rm = TRUE))

Object of class "tbl_dftbldata.frame"

group count mean sd median IQR

1 Blasto 11 5221.818 2747.830 5912 3968.5

2 E_coli 9 2221.667 3198.033 791 1756.0

3 E_nana 4 3670.000 3652.280 3033 3787.0

4 NPDM 25 2064.920 2008.508 1696 2950.0

> kruskal.test(sample_data(myData_CNSG)$PrevotellaceaeCount, sample_data(myData_CNSG)$group, pvalue.threshold = 0.05)

Kruskal-Wallis rank sum test

data: sample_data(myData_CNSG)$PrevotellaceaeCount and sample_data(myData_CNSG)$group

Kruskal-Wallis chi-squared = 10.274, df = 3, **p-value = 0.01638**

> pairwise.wilcox.test(sample_data(myData_CNSG)$PrevotellaceaeCount, sample_data(myData_CNSG)$group)

Pairwise comparisons using Wilcoxon rank sum test

data: sample_data(myData_CNSG)$PrevotellaceaeCount and sample_data(myData_CNSG)$group

Blasto E_coli E_nana

E_coli 0.201 - -

E_nana 1.000 0.986 -

NPDM **0.013** 1.000 0.986

P value adjustment method: holm

Warning messages:

1: In wilcox.test.default(xi, xj, paired = paired, ...) :

cannot compute exact p-value with ties

**Supplementary Figure 1. Top ten most abundant phyla.**

Bar plot representing the normalized top ten most abundant phyla per child in the groups NPDM (Controls with no parasites detected in the microscopic stool analysis), **Blasto** (Children parasitized only by *Blastocystis* spp.). **E_coli** (Children parasitized only by *Entamoeba coli*). **E_nana** (Children parasitized only by *Endolimax nana*)*.* The remaining Phyla were merged in the **Other** category.

**Supplementary Figure 2. Top ten most abundant bacterial Families.**

Bar plot representing the normalized top ten most abundant Families per child in the groups NPDM (Controls with no parasites detected in the microscopic stool analysis), **Blasto** (Children parasitized only by *Blastocystis* spp.). **E_coli** (Children parasitized only by *Entamoeba coli*). **E_nana** (Children parasitized only by *Endolimax nana*)*.* The remaining Families were merged in the **Other** category.

**Supplementary Figure 3. Top ten most abundant bacterial Genera.**

Bar plot representing the normalized top ten most abundant Genera per child in the groups **NPDM** (Controls with no parasites detected in the microscopic stool analysis), **Blasto** (Children parasitized only by *Blastocystis* spp.). **E_coli** (Children parasitized only by *Entamoeba coli*). **E_nana** (Children parasitized only by *Endolimax nana*)*.* The remaining Genera were merged in the **Other** category.

**Supplementary Table 1. General statistics of the processed reads and the calculated indices for each sample.**

| **sample** | **GROUP** | **nseqs** | **coverage** | **sobs** | **shannon** | **chao** |
| --- | --- | --- | --- | --- | --- | --- |
| 50kFAN_SE_009 | E_nana | 12440 | 0.993248 | 193 | 2.701194 | 461.153846 |
| 50kFAN_ED_012 | NPDM | 12464 | 0.990934 | 212 | 2.655145 | 545.052632 |
| 50kAA_064 | NPDM | 12374 | 0.992727 | 222 | 3.078946 | 315.139535 |
| 50kAA_001 | NPDM | 12387 | 0.994349 | 223 | 3.332672 | 292 |
| 50kRA_038 | NPDM | 12392 | 0.992899 | 230 | 3.08733 | 313.217391 |
| 50kSE_007 | NPDM | 12391 | 0.992091 | 242 | 2.87858 | 370.459459 |
| 50kCA_020 | NPDM | 12390 | 0.992494 | 244 | 3.165982 | 324.716981 |
| 50kCA_035 | NPDM | 12382 | 0.993216 | 246 | 3.409755 | 329 |
| 50kRA_17 | NPDM | 12402 | 0.993146 | 254 | 3.537123 | 343.25 |
| 50kAA_025 | NPDM | 12365 | 0.992155 | 270 | 3.708826 | 375.818182 |
| 50kCA_044 | NPDM | 12373 | 0.99014 | 271 | 3.162817 | 438.75 |
| 50kCA_032 | NPDM | 12367 | 0.991267 | 276 | 3.472004 | 445.941176 |
| 50kSE_017 | NPDM | 12379 | 0.992083 | 277 | 3.174811 | 382.622222 |
| 50kED_027 | NPDM | 12356 | 0.99304 | 281 | 2.908099 | 385.428571 |
| 50kED_003 | NPDM | 12326 | 0.990589 | 283 | 3.298208 | 428 |
| 50kRA_016 | NPDM | 12379 | 0.992649 | 291 | 3.438778 | 382 |
| 50kFAN_ED_029 | NPDM | 12452 | 0.985866 | 308 | 2.749329 | 821.333333 |
| 50kAA_013 | NPDM | 12358 | 0.991018 | 328 | 3.669478 | 463.666667 |
| 50kFAN_SE_015 | blasto | 12494 | 0.986554 | 333 | 2.32291 | 644.733333 |
| 50kSE_035 | NPDM | 12357 | 0.991017 | 347 | 3.750429 | 476.893617 |
| 50kFAN_ES_013 | blasto | 12468 | 0.984921 | 348 | 3.030899 | 999.037037 |
| 50kFAN_ES_005 | NPDM | 12476 | 0.984049 | 365 | 3.395283 | 1044.344828 |
| 50kFAN_ES_007 | NPDM | 12480 | 0.989824 | 369 | 3.493729 | 475.68 |
| 50kFAN_RA_063 | E_coli | 12489 | 0.986949 | 378 | 3.106247 | 518.457447 |
| 50kFAN_ES_029 | blasto | 12466 | 0.982031 | 381 | 3.089274 | 1186.677419 |
| 50kFAN_ES_002 | NPDM | 12495 | 0.982313 | 385 | 3.369592 | 1008.333333 |
| 50kFAN_CO_018 | NPDM | 12454 | 0.982255 | 391 | 3.418642 | 1030.736842 |
| 50kFAN_ED_008 | blasto | 12511 | 0.981137 | 395 | 2.382472 | 985 |
| 50kFAN_ED_014 | blasto | 12493 | 0.98215 | 395 | 2.854768 | 1168.53125 |
| 50kFAN_ES_028 | E_coli | 12481 | 0.981332 | 400 | 3.548639 | 1271.870968 |
| 50kFAN_CA_009 | E_coli | 12493 | 0.986312 | 407 | 4.008864 | 600.8 |
| 50kFAN_SE_008 | blasto | 12508 | 0.986329 | 413 | 3.385562 | 555.5 |
| 50kFAN_CO_007 | E_nana | 12517 | 0.980267 | 419 | 2.127541 | 1218.5 |
| 50kFAN_ES_027 | blasto | 12477 | 0.982287 | 420 | 3.350716 | 1230.333333 |
| 50kFAN_ED_037 | E_nana | 12497 | 0.985036 | 431 | 3.402199 | 716.098361 |
| 50kFAN_AA_023 | E_coli | 12510 | 0.984333 | 445 | 3.553624 | 642.010309 |
| 50kFAN_ES_001 | blasto | 12483 | 0.978851 | 457 | 2.546696 | 1324.9 |
| 50kFAN_CO_022 | blasto | 12468 | 0.980671 | 473 | 3.031395 | 1063.204082 |
| 50kFAN_AA_004 | E_coli | 12493 | 0.983751 | 473 | 3.397442 | 676 |
| 50kFAN_RA_54 | NPDM | 12510 | 0.977138 | 474 | 2.918805 | 1672.676471 |
| 50kFAN_AA_024 | E_coli | 12518 | 0.984422 | 476 | 3.717147 | 712.4375 |
| 50kFAN_ES_019 | blasto | 12437 | 0.978451 | 479 | 3.487506 | 1256.782609 |
| 50kFAN_ED_073 | E_coli | 12520 | 0.986022 | 481 | 3.586157 | 585.280822 |
| 50kFAN_AA_014 | E_coli | 12497 | 0.986077 | 492 | 4.221393 | 677.814815 |
| 50kFAN_ED_036 | NPDM | 12510 | 0.984492 | 496 | 3.277119 | 641.124031 |
| 50kFAN_RA_56 | NPDM | 12476 | 0.975713 | 500 | 3.42022 | 1770.916667 |
| 50kFAN_ED_013 | blasto | 12526 | 0.97581 | 518 | 2.496044 | 1162.408451 |
| 50kFAN_RA_058 | E_nana | 12443 | 0.982721 | 533 | 3.76458 | 852.513889 |
| 50kFAN_ES_025 | E_coli | 12476 | 0.97411 | 551 | 2.877766 | 1919.5 |
